# Predictive Accuracy of Biochemical and Anthropometric Indices for Metabolic Syndrome in Children with Obesity: A Comparative Study

**DOI:** 10.3390/life15020216

**Published:** 2025-01-31

**Authors:** Cihad Dundar

**Affiliations:** Department of Public Health, Faculty of Medicine, Ondokuz Mayis University, Samsun 55270, Türkiye; cdundar@omu.edu.tr

**Keywords:** cardio metabolic index, childhood obesity, homeostasis model assessment of insulin resistance, metabolic syndrome, triglyceride–glucose index, visceral adiposity index

## Abstract

Children with obesity, regardless of gender, are a high-risk population that requires ongoing monitoring not only for present obesity and metabolic syndrome (MetS) but also future risks of metabolic, cardiac, musculoskeletal, and psychiatric complications. Data from a cohort of 185 obese children who underwent a second follow-up in 2019 were used for this retrospective study. The study cohort consisted of 94 boys and 91 girls who were elementary school students with a mean age of 10.2 ± 0.5 years. Following anthropometric and biochemical assessments, the cardio metabolic index (CMI), visceral adiposity index (VAI), triglyceride–glucose index (TyGI), and homeostasis model assessment of insulin resistance (HOMA-IR) were calculated. The overall prevalence of MetS was 19.5% (12.8% in boys and 26.4% in girls). According to the receiver operating characteristic curve analysis, CMI, VAI, and TyGI performed significantly better than HOMA-IR in identifying MetS. CMI was the most accurate predictor of MetS, as indicated by the highest area under the curve value, in both genders. In conclusion, our findings suggest that the CMI can serve as a practical, efficient, and affordable screening tool for the ongoing monitoring of childhood obesity in both daily endocrine clinical practice and primary care settings.

## 1. Introduction

Childhood obesity has become a global epidemic; obese children carry cardio metabolic and psychosocial complications to adolescent and adulthood age [1]. The World Health Organization (WHO) declared that overweight and obesity rates among European primary school children range from 13% to 52%, with southern European countries experiencing particularly high levels [2]. If current trends persist, the global count of overweight or obese children is expected to peak at a staggering 70 million by 2025 [3]. Children with obesity are at a higher risk of remaining overweight or obese as adults, increasing their likelihood of developing serious health problems [4]. These problems create a growing burden on both the socioeconomic system and health services with a wide pattern, ranging from premature death to chronic diseases such as metabolic syndrome (MetS), cardiovascular diseases, and cancer [5]. A cohort study found that children aged 8–10 years with MetS and overweight were 3.21 times more likely to develop MetS in adulthood [6].

MetS is an umbrella term that encompasses a series of anthropometric and biochemical abnormalities occurring together in the same individual [7]. MetS, characterized by a cluster of risk factors including excess abdominal adiposity, high triglycerides, elevated blood pressure, high blood glucose, and low high-density lipoprotein cholesterol (HDL-C), significantly increases the risk of developing type 2 diabetes, cardiovascular disease, and chronic systemic inflammation [8]. The lack of a standardized MetS definition for children makes it difficult to accurately determine the prevalence of MetS in this population [9]. However, it significantly rises from 3.3% in the general population to 29.2% in obese children, highlighting MetS as a growing public health concern in the pediatric population [10].

Given its crucial role in the pathogenesis of MetS, there is a pressing need for the development of tools and indices that can reflect visceral adipose tissue expansion. While imaging techniques such as magnetic resonance imaging and computed tomography scans are considered the gold standard for measuring visceral adipose tissue volume, they are expensive and/or expose patients to high doses of radiation, rendering them less cost-effective options [11]. Since it is vital to identify patients with obesity at risk of developing MetS at an early stage, various anthropometric and biochemical indices, including waist circumference (WC), waist-to-hip ratio, body mass index (BMI), and homeostasis model assessment of insulin resistance (HOMA-IR), have been proposed for this purpose over the years [12]. Recently, several indices have gained prominence in predicting MetS. These include the visceral adiposity index (VAI), which combines anthropometric measures (WC and BMI) with biochemical factors [triglycerides (TG) and HDL-C]. The triglyceride–glucose index (TyGI) is another significant predictor, considered effective in assessing insulin resistance [13,14]. Finally, the cardiometabolic index (CMI), which integrates the waist-to-height ratio, an indicator of adiposity, with the TG/HDL-C ratio, has become more prominent in the prediction of MetS [15].

Indicators such as adiposity, blood lipid levels, and glucose markers have become essential tools for evaluating metabolic health. However, there is no general consensus on which of these indices has performed best so far [16]. When compared to traditional adiposity indicators, VAI has been identified as the best predictor of MetS in obese children in some studies [17,18], Likewise, TyGI has been shown to be a robust predictor of MetS in other studies [13,19]. However, studies comparing HOMA-IR with TyGI and VAI have consistently found HOMA-IR to be a less accurate predictor of MetS [20,21,22]. While the number of studies comparing MetS indicators in children is more limited, there is only one study investigating the predictive power of the CMI for MetS in children with obesity [23]. A thorough review of the literature did not yield any studies that have conducted a comparative analysis of the predictive accuracy of these indices for MetS in children with obesity. Therefore, we aimed to compare the ability of the four most commonly used indices, CMI, VAI, TyGI and HOMA-IR, to predict MetS in children with obesity by gender.

## 2. Materials and Methods

### 2.1. Study Design and Participants

The ‘Easier to Bend a Twig Than a Tree (EBTAT)’ project, a population-based longitudinal study focusing on children with obesity, has been conducted in Samsun, a major city situated on the Black Sea coast of Turkey. In 2016, a cross-sectional study was conducted on a sample of 9786 primary school students representing Samsun province and all its districts to determine the prevalence of obesity among primary school-aged children. Of the 1030 (10.5%) children identified as obese, 403 first- and second-grade students were followed up.

In 2019, students from the follow-up group who were reachable and agreed to participate were re-evaluated clinically, anthropometrically, and biochemically. During the follow-up period, 19 out of the 403 children were lost to follow-up. Subsequently, the anthropometric and clinical characteristics of the remaining participants were reassessed. Children who no longer met the criteria for obesity (n = 14) and those receiving medication for obesity or other metabolic conditions (n = 15) were excluded from further analysis. According to the WHO age- and sex-adjusted BMI tables, 355 children with a body mass index (BMI) greater than two standard deviations were included in the study. The n = (Nt^2^pq)/[d^2^(N − 1) + t^2^pq] formula was used to determine the minimum sample size. To ensure adequate statistical power, a sample size calculation was performed. Based on a target population of 355 children, an estimated MetS prevalence of 30%, a confidence level of 95% (corresponding to a Z-score of 1.96), and a desired margin of error of 5%, the required sample size was determined to be 169. To account for potential laboratory errors, the sample size was increased by 10% to 185 children. Participants were then selected using a systematic sampling method. The current retrospective study utilized data from the second follow-up EBTAT cohort, consisting of 185 children with obesity. Prior to any health assessments, including surveys, anthropometric measurements, and biochemical tests, written informed consent was obtained from the parents or legal guardians of all participants.

### 2.2. Measurements

Obesity was diagnosed based on a BMI-for-age criteria (greater than two standard deviations above the WHO reference median) [24]. Several definitions exist for MetS, including those from the International Diabetes Federation (IDF), the National Cholesterol Education Program Adult Treatment Panel III (NCEP ATP III), and the WHO [6,9]. These definitions typically encompass a combination of central obesity, elevated triglycerides, reduced HDL-cholesterol, elevated blood pressure, and elevated fasting glucose. While NCEP ATP III emphasizes the presence of at least three of these criteria, the WHO definition prioritizes insulin resistance. The IDF definition, chosen for this study, emphasizes abdominal obesity as a prerequisite, acknowledging its strong association with cardiometabolic risk [25,26]. It defines MetS as the presence of central obesity (waist circumference ≥90th percentile) plus at least two other criteria: TG ≥ 150 mg/dL, HDL-C < 40 mg/dL, systolic blood pressure ≥130 mmHg or diastolic blood pressure ≥85 mmHg, and fasting blood glucose (FBG) ≥ 100 mg/dL [27].

Children were measured in light clothing for waist circumference, height, and weight. To minimize errors in anthropometric measurements, standardized measurement procedures were implemented [28]. Calibrated scales and stadiometers were used, and personnel were trained in their proper use. Waist circumference was measured at the level of the umbilicus. Blood pressure was measured twice at five-minute intervals. Fasting blood samples were collected from all children between 8:00 a.m. and 9:00 a.m. Serum levels of total cholesterol (TC), low-density lipoprotein cholesterol (LDL-C), high-density lipoprotein cholesterol (HDL-C), triglycerides (TG), and fasting blood glucose (FBG) were subsequently measured. Biochemical analyses of fasting blood samples were performed using a COBAS 8000 c-702 (Roche Diagnostics, Indianapolis, IN, USA) autoanalyzer with colorimetric methods.

Insulin resistance was assessed using the HOMA-IR index formula: FBG (mmol/L) × fasting insulin (IU/mL)/405. In a meta-analysis study in which HOMA-IR cut-off points related to MetS in children and adolescents, the region of HOMA-IR associated with MetS was found to range from 2.30 to 3.54 [29]. Since all of the children in our study group were obese, a HOMA-IR value greater than 3.54, the upper value determined in this study, was considered an indicator of insulin resistance. The TyGI was calculated using the following formula: ln [(fasting TG (mg/dL) × FBG (mg/dL))/2] [30]. The VAI was calculated separately for boys and girls as follows [23]:

Boy = [WC∕(39.68 + (1.88 × BMI))] × (TG∕1.03) × (1.31∕HDL-C);

Girl = [WC∕(36.58 + (1.89 × BMI))] × (TG∕0.81) × (1.52∕HDL-C)

The CMI was measured using the following formula: (TG/HDL-C) × (WC/Height) [23].

### 2.3. Statistical Analysis

All statistical analyses were conducted using SPSS version 22.0 (IBM Corporation, Armonk, NY, USA). The normality of continuous variables was assessed using the Kolmogorov–Smirnov test. Data are presented as mean ± standard deviation (SD) for continuous variables and as frequencies and percentages for categorical variables. The categorical variables were compared by the Pearson Chi-square test. The Mann–Whitney U test was used to compare the quantitative data that were non-normally distributed, while data that were normally distributed were compared using Student’s *t*-test. We used the receiver operating characteristic (ROC) curve analyses to examine the sensitivity, specificity, and cut-off points of the indexes. The area under the curve (AUC) was calculated to evaluate the overall performance of each index (VAI, TyGI, CMI, and HOMA-IR) for predicting the development of MetS. The optimal cut-off points were determined as the points on the curve where both sensitivity and specificity were highest. Statistical significance was set at *p* < 0.05.

## 3. Results

The study cohort comprised 185 primary school students, all classified as obese, with a mean age of 10.2 ± 0.5 years. Gender-based comparisons of anthropometric measurements showed no significant differences, except for waist circumference. However, metabolic indices were notably higher among girls compared to boys, especially VAI and HOMA-IR, as presented in Table 1.

A total of 12 (12.8%) boys and 24 (26.4%) girls were found to have MetS. The overall prevalence of MetS in the cohort was 19.5%, and it was twice as high in girls compared to boys (*p* = 0.019; OR = 2.45, 95% CI = 1.14–5.26). Both boys and girls with MetS had significantly higher systolic and diastolic blood pressure and triglyceride levels. However, they had significantly lower HDL-C levels compared to those without MetS. (Table 2).

**Figure 1:**
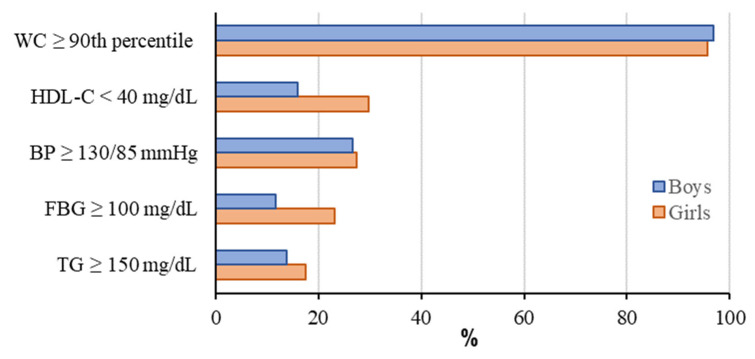
Gender disparities in metabolic risk profiles. WC: waist circumference; HDL-C: high-density lipoprotein cholesterol; BP: blood pressure; FBG: fasting blood glucose; TG: triglyceride.

Girls were found to have significantly higher levels of increased FBG and lower HDL-C, both components of MetS (*p* < 0.05) (Figure 1).

As a result of the ROC analysis, CMI demonstrated the largest and most significant AUC in predicting MetS in both boys and girls (Table 3). HOMA-IR, which ranked last in both genders, did not have a statistically significant AUC value in boys.

**Figure 2:**
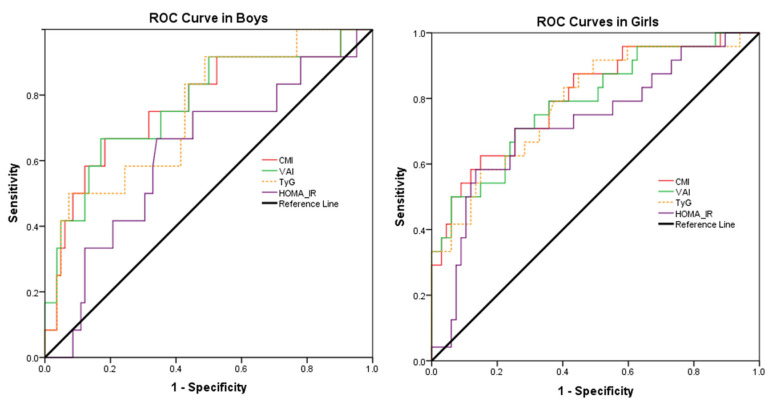
ROC curves of CMI, VAI, TyGI, and HOMA-IR to predict MetS in children with obesity by gender. CMI: cardio metabolic index; VAI: visceral adiposity index; TyGI: triglyceride glucose index; HOMA-IR: homeostatic model assessment for insulin resistance index.

Sensitivity and specificity values did not differ significantly among CMI, VAI, and TyGI. As presented in the ROC curve graphs, it was determined that in girls with obesity, all indicators, including HOMA-IR, covered a wider area compared to boys (Figure 2).

## 4. Discussion

Our study findings indicate that approximately one in seven boys with obesity meet the criteria for MetS. However, the even more striking finding is that this proportion increases to nearly one-quarter in girls with obesity. While some studies conducted in peri-pubertal children have found a higher prevalence of MetS in boys [31,32], others have reported a higher prevalence in girls [33,34]. Children with obesity, regardless of gender, represent a high-risk population that requires monitoring not only for current obesity and MetS but also for potential metabolic, cardiac, musculoskeletal, and psychiatric disorders that they may face in the near future and adulthood [35]. Therefore, screening programs play a vital role in identifying children who may be at elevated risk.

We conducted a direct comparison of CMI, VAI, TyGI, and HOMA-IR indices to determine their ability to predict MetS in children with obesity. Although HOMA-IR exhibited the lowest AUC among all evaluated indicators in both genders, CMI, VAI, and TyGI demonstrated considerably superior performance. Among all the indicators, CMI demonstrated the highest and most significant AUC in predicting MetS in both boys and girls. A comparative analysis of BMI, Tri-Ponderal Mass Index, waist-to-height ratio, TG/HDL-C ratio, CMI, and VAI for predicting MetS in Italian children and adolescents with obesity revealed that VAI, CMI, and TG/HDL-C ratio were significantly better predictors [23]. Research conducted in both Romania and Mexico has shown that the TG/HDL-C ratio is a robust predictor of MetS in comparable age groups [36,37].

Our findings are consistent with previous studies that have demonstrated the high sensitivity and specificity of CMI, TyGI, and VAI in the detection of MetS among children with obesity [16,23]. Lazzer [16] postulated that VAI and CMI, owing to their superior sensitivity and specificity, may offer greater clinical utility. Our results support the high sensitivity of CMI, with values exceeding those reported by Laser [16] and Radetti [23] (0.75 vs. 0.68 and 0.72), albeit with a slightly lower specificity (0.65 vs. 0.87 and 0.84). The heterogeneity in sensitivity and specificity among the indices can be attributed to the diverse populations studied, which varied in terms of ethnicity and pubertal status. Furthermore, the lack of a standardized definition of MetS for children under ten years of age may account for some of the observed variability.

It has been demonstrated that CMI, which is associated with an increased risk of cardiovascular disease, chronic kidney disease, and non-alcoholic fatty liver disease in the adult population, is one of the most effective predictors of MetS when compared to other anthropometric indices [16,38,39]. The high predictive values of CMI and VAI can be attributed to their formulas, which incorporate both anthropometric and biochemical parameters. However, the VAI formula incorporates the BMI as an anthropometric assessment measure, while the CMI formula utilizes the waist-to-height ratio. Several studies reported that the waist-to-height ratio has higher discriminating ability than BMI in detecting high body fat percentages among children [40,41,42]. It is hypothesized that CMI’s ability to more accurately assess body fat percentage contributes to its status as the MetS indicator with the highest AUC in children with obesity. A recent study conducted in China revealed that the CMI has a strong predictive ability in identifying metabolically abnormal phenotypes [43]. A comparative study conducted in Italian children with obesity reported that both the VAI and CMI exhibited the best performance in predicting MetS, and there was no significant difference between the two indices [23].

When we evaluated the ROC curves for boys and girls with obesity, we found that the AUC covered a larger area in girls. The indicators used in the study (including HOMA-IR) are generally better at distinguishing between obese girls with and without MetS. The results indicate that the indices used to predict MetS were more accurate in girls with obesity than in boys with obesity. This could imply that there are gender-specific differences in the development or manifestation of MetS in children. While z-BMI is commonly used to classify childhood obesity in most studies, other measures of adiposity, such as waist circumference or skin fold thickness, are often overlooked or under-investigated. Furthermore, limited research has explored gender-specific variations in the overweight/adiposity metabolome in children with obesity. Most of these have been conducted with older children and adolescents, for whom puberty changes become an important confounder. A substantial portion of adult body weight, estimated to be around 50%, is acquired during the pubertal growth phase. While lean mass and skeletal mass are high in boys, fat mass increases in girls. Prior to puberty, girls typically exhibit higher serum follicle-stimulating hormone (FSH) levels compared to boys. Studies have shown that elevated serum FSH levels in obese prepubertal children are associated with an increased risk of developing metabolic syndrome (MetS) during the pubertal transition. It has also been shown that an increase in serum FSH in the prepubertal years is associated with an increased likelihood of BMI elevation during follow-up [44]. Girls were found to be more insulin resistant than boys at all Tanner stages and BMI increased throughout puberty [45]. Disturbances in all these physiological changes in adolescence may contribute to the development of cardiovascular disease and other adult-onset health problems.

Azab et al. [46] showed that metabolic differences can arise depending on the choice of obesity or adiposity measure and demonstrated the specificity and additive value of waist circumference measurement in predicting morbidity. The association between some serum metabolites and overweight/adiposity were found to only be significant in females. While most metabolites indicated nearly twofold greater estimates of overweight/adiposity among females, the corresponding odds ratios for males were not significant. When the analysis was stratified by gender, it was observed that the metabolic profile associated with overweight and adiposity at age five years was more pronounced in girls. In contrast, the associations between these factors and the metabolic profile were weaker and often non-significant in boys [46]. The serum levels of tyrosine and phenylalanine have been found to be positively associated with z-BMI in girls only, whereas in boys, significant associations with serum levels of leucine and isoleucine have been seen post-puberty [47]. Sex-specific differences may be due to earlier maturation in girls or differences in mitochondrial plasticity between boys and girls [46]. Apart from hormonal factors, genetic factors, differences in lifestyle and dietary patterns have also been evaluated as potential mechanisms underlying sex-related variation. These highlight the need for prospective studies and more gender-specific analyses to explain the relationship between obesity, cardiometabolic disease, and gender.

The retrospective nature of the study and its single-center design represent potential limitations. First, the use of single-timepoint glucose and triglyceride measurements is limited by its inability to fully capture metabolic variability, which could introduce bias into the AUC results. Therefore, a longitudinal study design is required to evaluate the evolving predictive capabilities of these different indexes for MetS in children with obesity. Second, the information about the comorbidities of each participant was not available, thus limiting our ability to assess their frequency and their potential correlations with the assessed indices. Third, the study sample consisted entirely of Caucasian children with obesity, which may not fully reflect the experiences of children from other ethnic backgrounds. Finally, our preclinical investigation, grounded in biochemical and anthropometric measurements, focused on a comparative analysis of the diagnostic performance of several indices for MetS using ROC curve analysis. Our study’s primary focus on the statistical performance of the indices limited its ability to directly assess clinical outcomes.

## 5. Conclusions

Further research is needed to investigate the optimal cut-off points and the long-term efficacy and cost-effectiveness of CMI for MetS detection in different populations and to explore its potential for identifying children at high risk for specific MetS-related complications. To determine if the differences observed in ROC analysis translate into clinically significant outcomes, such as the development of type 2 diabetes, cardiovascular disease, or other metabolic complications, larger sample sizes and longer follow-up periods are necessary. Also, we must determine whether the use of the CMI as a screening tool leads to earlier intervention and improved treatment outcomes, and whether it is more cost-effective than other methods in different clinical settings. The use of CMI for MetS detection in children with obesity could provide a simple, fast, and cost-effective screening method in everyday endocrine clinical practice, and in resource-limited settings such as primary health care.

## Figures and Tables

**Table 1:** Gender differences in anthropometric and biochemical characteristics of the children with obesity (mean ± SD).

Characteristics	Boys (n = 94)	Girls (n = 91)	*p*
Age (Year)	10.2 ± 0.5	10.2 ± 0.5	0.942
Height (cm)	143.1 ± 6.4	144.8 ± 6.9	0.098
Weight (kg)	56.3 ± 8.1	56.5 ± 7.8	0.887
WC (cm)	84.4 ± 8.2	81.2 ± 7.7	0.003
HC (cm)	94.7 ± 6.6	95.3 ± 6.9	0.611
BMI (kg/m^2^)	27.4 ± 2.3	26.9 ± 2.3	0.140
Systolic BP (mmHg)	116.6 ± 10.2	118.0 ± 11.1	0.380
Diastolic BP (mmHg)	73.6 ± 8.4	74.0 ± 8.9	0.764
Insulin (µIU/mL)	17.9 ± 5.1	21.4 ± 7.4	0.001
FBG (mg/dL)	90.4 ± 14.8	92.2 ± 13.5	0.381
TC (mg/dL)	151.0 ± 29.5	147.4 ± 30.1	0.408
TG (mg/dL)	105.2 ± 58.6	109.6 ± 51.7	0.590
LDL-C (mg/dL)	81.2 ± 26.4	80.2 ± 26.2	0.794
HDL-C (mg/dL)	48.8 ± 11.1	45.3 ± 8.4	0.037
CMI	1.42 ± 1.12	1.45 ± 0.88	0.823
VAI	2.81 ± 2.09	4.38 ± 2.67	<0.001
TyGI	8.25 ± 0.82	8.39 ± 0.67	0.219
HOMA-IR	4.01 ± 1.32	4.94 ± 2.10	0.002

BP: blood pressure; CMI: cardio metabolic index; FBG: fasting blood glucose; HC: hip circumference; HDL-C: high-density lipoprotein cholesterol; HOMA-IR: homeostatic model assessment for insulin resistance index; IU: international unit; LDL-C: low-density lipoprotein cholesterol; TC: total cholesterol; TG: triglyceride; TyGI: triglyceride glucose index; VAI: visceral adiposity index; WC: waist circumference.

**Table 2:** Characteristics of the children by gender (mean ± SD).

	Boys			Girls		
Characteristics	MetS (n = 12)	Non-MetS (n = 82)	*p*	MetS (n = 24)	Non-MetS (n = 67)	*p*
WC (cm)	89.0 ± 8.8	83.7 ± 7.9	0.039	83.6 ± 6.1	80.7 ± 8.2	0.188
HC (cm)	98.9 ± 6.5	94.1 ± 6.5	0.016	94.9 ± 6.3	95.4 ± 7.2	0.811
BMI (kg/m2)	28.7 ± 2.6	27.2 ± 2.3	0.062	27.2 ± 2.6	26.8 ± 2.2	0.620
Systolic BP (mmHg)	125.9 ± 8.8	115.3 ± 9.7	0.001	124.2 ± 9.6	115.8 ± 10.8	0.002
Diastolic BP (mmHg)	81.9 ± 7.5	72.4 ± 7.9	<0.001	80.5 ± 8.3	71.7 ± 8.1	<0.001
Insulin (µIU/mL)	18.4 ± 4.0	17.9 ± 5.2	0.424	24.6 ± 7.8	20.3 ± 7.0	0.010
FBG (mg/dL)	95.7 ± 7.4	89.6 ± 15.5	0.089	99.9 ± 10.6	89.4 ± 13.4	<0.001
TC (mg/dL)	160.9 ± 35.4	149.6 ± 28.5	0.350	154.2 ± 30.5	144.9 ± 29.8	0.202
TG (mg/dL)	155.3 ± 84.3	97.9 ± 50.5	0.016	146.3 ± 69.8	96.5 ± 35.8	<0.001
LDL-C (mg/dL)	90.4 ± 32.1	79.8 ± 25.5	0.522	85.4 ± 26.0	78.3 ± 26.2	0.251
HDL-C (mg/dL)	40.3 ± 6.9	50.1 ± 11.0	<0.001	39.5 ± 7.3	47.3 ± 7.9	<0.001
CMI	2.63 ± 2.10	1.25 ± 0.77	0.003	2.22 ± 1.19	1.18 ± 0.53	<0.001
VAI	5.11 ± 3.70	2.48 ± 1.50	0.002	6.65 ± 3.67	3.56 ± 1.58	<0.001
TyGI	8.77 ± 0.52	8.18 ± 0.83	0.006	8.79 ± 0.48	8.24 ± 0.68	<0.001
HOMA-IR	4.36 ± 1.07	3.96 ± 1.36	0.165	6.15 ± 2.43	4.51 ± 1.80	0.001

BP: blood pressure; CMI: cardio metabolic index; HC: hip circumference; HDL-C: high-density lipoprotein cholesterol; HOMA-IR: Homeostatic model assessment for insulin resistance index; IU: International unit; LDL-C: low-density lipoprotein cholesterol; TC: total cholesterol; TyGI: triglyceride glucose index; VAI: visceral adiposity index; WC: waist circumference.

**Table 3:** Cut-off values of CMI, VAI, TyGI, and HOMA-IR to predict MetS in children with obesity by gender.

Gender/Index	AUC (95% CI)	*p*	Cut-Off Value	Sensitivity–Specificity
Boy				
CMI	0.771 (0.616.0.927)	0.003	1.455	0.75–0.65
VAI	0.770 (0.615–0.926)	0.002	2.724	0.75–0.68
TyGI	0.749 (0.603–0.895)	0.006	8.322	0.75–0.57
HOMA-IR	0.624 (0.454–0.795)	0.165	3.922	0.75–0.55
Girl				
CMI	0.805 (0.700–0.910)	<0.001	1.291	0.79–0.64
TyGI	0.788 (0.680–0.896)	<0.001	8.427	0.79–0.63
VAI	0.787 (0.678–0.897)	<0.001	4.077	0.79–0.64
HOMA-IR	0.725 (0.601–0.849)	0.001	4.382	0.75–0.57

AUC: area under curve; CMI: cardio metabolic index; VAI: visceral adiposity index; TyGI: triglyceride glucose index; HOMA-IR: homeostatic model assessment for insulin resistance index.

## Data Availability

The data that support the findings of this study are available on request from the author.
